# 5-[(*E*)-4-Fluoro­benzyl­idene]-8-(4-fluoro­phen­yl)-2-hy­droxy-9-phenyl-3,10-diaza­hexa­cyclo­[10.7.1.1^3,7^.0^2,11^.0^7,11^.0^16,20^]henicosa-1(20),12,14,16,18-pentaen-6-one

**DOI:** 10.1107/S1600536811040633

**Published:** 2011-10-08

**Authors:** Raju Suresh Kumar, Hasnah Osman, A. S. Abdul Rahim, Madhukar Hemamalini, Hoong-Kun Fun

**Affiliations:** a School of Chemical Sciences, Universiti Sains Malaysia, 11800 USM, Penang, Malaysia; b School of Pharmaceutical Sciences, Universiti Sains Malaysia, 11800 USM, Penang, Malaysia; c X-ray Crystallography Unit, School of Physics, Universiti Sains Malaysia, 11800 USM, Penang, Malaysia

## Abstract

In the title compound, C_38_H_28_F_2_N_2_O_2_, the piperidine ring adopts a chair conformation and the pyrrolidine ring adopts an envelope conformation with the spiro C atom as the flap atom. The naphthalene ring system makes dihedral angles of 39.89 (8), 35.33 (8) and 46.45 (8)° with the two fluoro-substituted benzene rings and the phenyl ring, respectively, while the dihedral angle between the two fluoro-substituted benzene rings is 75.21 (10)°. An intra­molecular O—H⋯N hydrogen bond generates an *S*(5) ring. In the crystal, mol­ecules are connected by C—H⋯O hydrogen bonds, forming supra­molecular chains propagating along the *c*-axis direction. Weak C—H⋯π inter­actions further consolidate the structure.

## Related literature

For further details of 1,3-dipolar cyclo­addition, see: Suresh Kumar *et al.* (2011 ▶); Jayashankaran *et al.* (2005 ▶); Manian *et al.* (2006 ▶); Williams & Fegley (1992 ▶). For ring conformations, see: Cremer & Pople (1975 ▶).
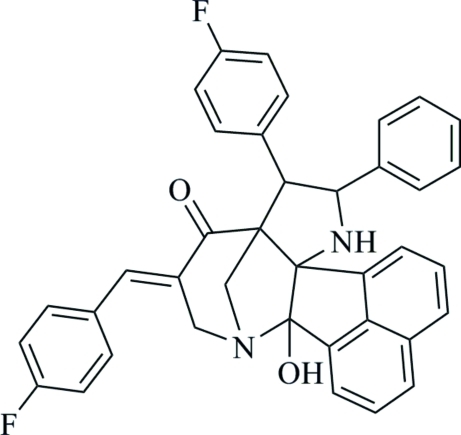


## Experimental

### 

#### Crystal data

C_38_H_28_F_2_N_2_O_2_
*M* *_r_* = 582.62
Triclinic, 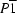

*a* = 9.3269 (3) Å
*b* = 11.8635 (4) Å
*c* = 14.2095 (4) Å
α = 75.904 (2)°
β = 74.726 (2)°
γ = 77.627 (2)°
*V* = 1452.01 (8) Å^3^
*Z* = 2
Mo *K*α radiation
μ = 0.09 mm^−1^
*T* = 296 K
0.39 × 0.25 × 0.15 mm

#### Data collection

Bruker SMART APEXII CCD diffractometer
Absorption correction: multi-scan (*SADABS*; Bruker, 2009 ▶) *T*_min_ = 0.965, *T*_max_ = 0.987
16981 measured reflections
5915 independent reflections
4221 reflections with *I* > 2σ(*I*)
*R*_int_ = 0.033

#### Refinement

*R*[*F* ^2^ > 2σ(*F* ^2^)] = 0.044
*wR*(*F* ^2^) = 0.115
*S* = 1.03
5915 reflections
405 parameters
H atoms treated by a mixture of independent and constrained refinement
Δρ_max_ = 0.17 e Å^−3^
Δρ_min_ = −0.20 e Å^−3^

### 

Data collection: *APEX2* (Bruker, 2009 ▶); cell refinement: *SAINT* (Bruker, 2009 ▶); data reduction: *SAINT*; program(s) used to solve structure: *SHELXTL* (Sheldrick, 2008 ▶); program(s) used to refine structure: *SHELXTL*; molecular graphics: *SHELXTL*; software used to prepare material for publication: *SHELXTL* and *PLATON* (Spek, 2009 ▶).

## Supplementary Material

Crystal structure: contains datablock(s) global, I. DOI: 10.1107/S1600536811040633/hb6426sup1.cif

Structure factors: contains datablock(s) I. DOI: 10.1107/S1600536811040633/hb6426Isup2.hkl

Supplementary material file. DOI: 10.1107/S1600536811040633/hb6426Isup3.cml

Additional supplementary materials:  crystallographic information; 3D view; checkCIF report

## Figures and Tables

**Table 1:** Hydrogen-bond geometry (Å, °) *Cg*5, *Cg*6, *Cg*7 and *Cg*9 are the centroids of the C1–C5/C10, C5–C10, C22–C27 and C34–C39 rings, respectively.

*D*—H⋯*A*	*D*—H	H⋯*A*	*D*⋯*A*	*D*—H⋯*A*
O2—H1*O*2⋯N2	0.90 (2)	1.97 (2)	2.636 (2)	129.6 (17)
C13—H13*A*⋯O2^i^	0.98	2.53	3.499 (2)	171
C20—H20*B*⋯O2^i^	0.97	2.55	3.522 (2)	179
C23—H23*A*⋯O1^ii^	0.93	2.52	3.410 (2)	162
C35—H35*A*⋯O2^i^	0.93	2.46	3.383 (2)	174
C30—H30*A*⋯*Cg*5^iii^	0.93	2.94	3.836 (2)	168
C31—H31*A*⋯*Cg*6^iii^	0.93	2.91	3.609 (3)	133
C38—H38*A*⋯*Cg*7^iv^	0.93	2.92	3.836 (2)	168
C7—H7*A*⋯*Cg*9^v^	0.93	2.85	3.400 (3)	119

Symmetry codes: (i) 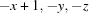; (ii) 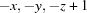; (iii) 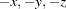; (iv) 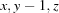; (v) 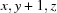.

**Fig. 1.**
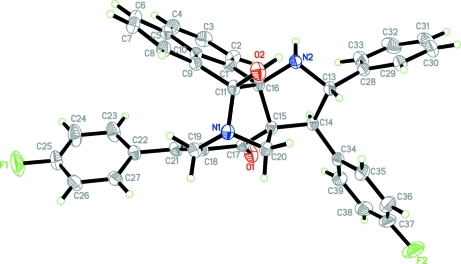
The asymmetric unit of the title compound, showing 30% probability displacement ellipsoids.

**Fig. 2.**
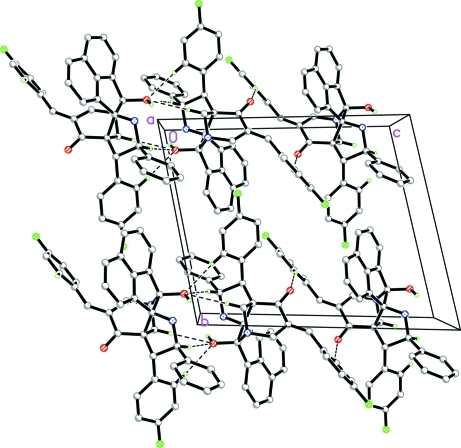
The crystal packing of the title compound (I). H atoms not involved in hydrogen bonding are omitted.

## supplementary crystallographic information

### Comment

1,3-Dipolar cycloaddition provides a facile route for the synthesis of many dispiroheterocyclic systems through the cycloaddition reaction of azomethine ylides with the definite dipolarophiles (Suresh Kumar *et al.*, 2011; Jayashankaran *et al.*, 2005; Manian *et al.*, 2006). This method is widely used as one of the key steps for the synthesis of natural products such as alkaloids and pharmacologically important compounds (Williams & Fegley, 1992). The significance of these heterocycles prompted us to investigate the crystal structure determination of the title compound and report the results in this paper.

The asymmetric unit of the title compound is shown in Fig. 1. The piperidine (N1/C15/C17–C20) ring adopts a chair conformation [Q = 0.6152 (17) Å; θ = 39.34 (16)° and φ = 298.6 (2)°; Cremer & Pople, 1975] and the pyrrolidine (N2/C13–C16) ring adopts an envelope conformation with the spiro C14 atom as the flap atom (displacement -0.230 (2) Å) and with puckering parameters, Q = 0.3614 (17) Å; and φ = 252.3 (3)°. The naphthalene (C1–C10) ring makes dihedral angles of 39.89 (8)°, 35.33 (8)° and 46.45 (8)°  with the two fluoro-substituted (C22–C27)/(C34–C39) phenyl rings and the benzene (C28–C33) ring, repectively. The corresponding angle between the two fluoro-substituted phenyl (C22–C27)/(C34–C39) rings is 75.21 (10)°.

In the crystal, (Fig. 2), the molecules are connected *via* C—H···O (Table 1) hydrogen bonds forming one-dimensional supramolecular chains along the *c*-axis. Furthermore, the crystal structure is stabilized by weak C—H···π interactions involving the centroids of the Cg5 (C1–C5/C10); Cg6 (C5–C10); Cg7 (C22–C27) and Cg9 (C34–C39) rings.

### Experimental

A mixture of 3,5-bis[(*E*)-(4-fluorophenyl)methylidene]tetrahydro-4 (1*H*)-pyridinone (1 mmol), acenaphthenequinone (1 mmol), and phenyl glycine (1 mmol) were dissolved in methanol (5 mL) and refluxed in a water bath for 1 hour. After completion of the reaction as evident from TLC, the mixture was poured into water (50 mL). The precipitated solid was filtered and washed with water to obtain the product which was further purified by recrystallisation from ethyl acetate to yield colourless blocks.

### Refinement

Atoms H1O2 and H1N2 were located from a difference Fourier maps and refined freely [N–H = 0.91 (2) Å and O–H = 0.90 (2) Å]. The remaining H atoms were positioned geometrically [C–H = 0.93–0.98 Å] and were refined using a riding model, with *U*_iso_(H) = 1.2 *U*_eq_(C).

### Crystal data

**Table:**

C_38_H_28_F_2_N_2_O_2_	*Z* = 2
*M**_r_* = 582.62	*F*(000) = 608
Triclinic, *P*1	*D*_x_ = 1.333 Mg m^−^^3^
Hall symbol: -P 1	Mo *K*α radiation, λ = 0.71073 Å
*a* = 9.3269 (3) Å	Cell parameters from 4467 reflections
*b* = 11.8635 (4) Å	θ = 2.6–27.1°
*c* = 14.2095 (4) Å	µ = 0.09 mm^−^^1^
α = 75.904 (2)°	*T* = 296 K
β = 74.726 (2)°	Block, colourless
γ = 77.627 (2)°	0.39 × 0.25 × 0.15 mm
*V* = 1452.01 (8) Å^3^	

### Data collection

**Table:**

Bruker SMART APEXII CCD diffractometer	5915 independent reflections
Radiation source: fine-focus sealed tube	4221 reflections with *I* > 2σ(*I*)
graphite	*R*_int_ = 0.033
φ and ω scans	θ_max_ = 26.5°, θ_min_ = 1.8°
Absorption correction: multi-scan (*SADABS*; Bruker, 2009)	*h* = −11→11
*T*_min_ = 0.965, *T*_max_ = 0.987	*k* = −14→14
16981 measured reflections	*l* = −17→17

### Refinement

**Table:**

Refinement on *F*^2^	Primary atom site location: structure-invariant direct methods
Least-squares matrix: full	Secondary atom site location: difference Fourier map
*R*[*F*^2^ > 2σ(*F*^2^)] = 0.044	Hydrogen site location: inferred from neighbouring sites
*wR*(*F*^2^) = 0.115	H atoms treated by a mixture of independent and constrained refinement
*S* = 1.03	*w* = 1/[σ^2^(*F*_o_^2^) + (0.0505*P*)^2^ + 0.1929*P*] where *P* = (*F*_o_^2^ + 2*F*_c_^2^)/3
5915 reflections	(Δ/σ)_max_ < 0.001
405 parameters	Δρ_max_ = 0.17 e Å^−^^3^
0 restraints	Δρ_min_ = −0.20 e Å^−^^3^

### Special details

**Table:**

Geometry. All s.u.'s (except the s.u. in the dihedral angle between two l.s. planes) are estimated using the full covariance matrix. The cell s.u.'s are taken into account individually in the estimation of s.u.'s in distances, angles and torsion angles; correlations between s.u.'s in cell parameters are only used when they are defined by crystal symmetry. An approximate (isotropic) treatment of cell s.u.'s is used for estimating s.u.'s involving l.s. planes.
Refinement. Refinement of F^2^ against ALL reflections. The weighted R-factor wR and goodness of fit S are based on F^2^, conventional R-factors R are based on F, with F set to zero for negative F^2^. The threshold expression of F^2^ > 2σ(F^2^) is used only for calculating R-factors(gt) etc. and is not relevant to the choice of reflections for refinement. R-factors based on F^2^ are statistically about twice as large as those based on F, and R- factors based on ALL data will be even larger.

### Fractional atomic coordinates and isotropic or equivalent isotropic displacement parameters (Å^2^)

**Table:**

	*x*	*y*	*z*	*U*_iso_*/*U*_eq_	
F1	0.31463 (18)	0.41565 (14)	0.63374 (12)	0.1021 (5)	
F2	0.56249 (16)	−0.63672 (10)	0.27077 (12)	0.0887 (4)	
O1	0.19391 (15)	−0.13595 (11)	0.41318 (8)	0.0499 (3)	
O2	0.40049 (15)	0.12921 (11)	0.03939 (8)	0.0451 (3)	
N1	0.46807 (15)	0.06737 (11)	0.19282 (9)	0.0358 (3)	
N2	0.17795 (18)	0.00560 (12)	0.10268 (9)	0.0376 (3)	
C1	0.08709 (18)	0.11620 (13)	0.24038 (10)	0.0338 (3)	
C2	−0.0512 (2)	0.09298 (16)	0.29527 (12)	0.0451 (4)	
H2A	−0.0774	0.0189	0.3052	0.054*	
C3	−0.1538 (2)	0.18431 (18)	0.33664 (14)	0.0553 (5)	
H3A	−0.2463	0.1678	0.3765	0.066*	
C4	−0.1225 (2)	0.29506 (18)	0.32049 (14)	0.0556 (5)	
H4A	−0.1936	0.3526	0.3486	0.067*	
C5	0.0179 (2)	0.32359 (15)	0.26106 (12)	0.0445 (4)	
C6	0.0639 (3)	0.43508 (16)	0.22979 (15)	0.0567 (5)	
H6A	−0.0006	0.5002	0.2507	0.068*	
C7	0.2025 (3)	0.44862 (16)	0.16899 (16)	0.0583 (5)	
H7A	0.2288	0.5236	0.1481	0.070*	
C8	0.3068 (2)	0.35270 (15)	0.13694 (13)	0.0477 (4)	
H8A	0.4013	0.3636	0.0970	0.057*	
C9	0.26533 (19)	0.24316 (14)	0.16619 (11)	0.0370 (4)	
C10	0.12161 (19)	0.23011 (13)	0.22507 (11)	0.0359 (4)	
C11	0.34421 (18)	0.12516 (13)	0.14237 (10)	0.0344 (4)	
C13	0.23219 (19)	−0.12206 (13)	0.10589 (11)	0.0350 (4)	
H13A	0.3328	−0.1310	0.0621	0.042*	
C14	0.24873 (18)	−0.17285 (13)	0.21430 (10)	0.0333 (3)	
H14A	0.1473	−0.1791	0.2554	0.040*	
C15	0.30126 (17)	−0.07313 (13)	0.24017 (10)	0.0316 (3)	
C16	0.21783 (18)	0.04178 (13)	0.18294 (10)	0.0325 (3)	
C17	0.26517 (18)	−0.06749 (13)	0.34904 (11)	0.0343 (4)	
C18	0.31892 (18)	0.03074 (13)	0.37188 (11)	0.0345 (4)	
C19	0.44652 (19)	0.08607 (15)	0.29507 (11)	0.0381 (4)	
H19A	0.4271	0.1701	0.2934	0.046*	
H19B	0.5397	0.0543	0.3171	0.046*	
C20	0.46882 (18)	−0.05780 (14)	0.19841 (12)	0.0373 (4)	
H20A	0.5312	−0.1069	0.2426	0.045*	
H20B	0.5058	−0.0778	0.1331	0.045*	
C21	0.2415 (2)	0.06910 (14)	0.45470 (11)	0.0397 (4)	
H21A	0.1604	0.0311	0.4903	0.048*	
C22	0.26546 (19)	0.16222 (15)	0.49779 (11)	0.0397 (4)	
C23	0.1410 (2)	0.23249 (19)	0.54396 (15)	0.0630 (6)	
H23A	0.0448	0.2219	0.5445	0.076*	
C24	0.1579 (3)	0.3180 (2)	0.58917 (18)	0.0773 (7)	
H24A	0.0740	0.3656	0.6192	0.093*	
C25	0.2994 (3)	0.33166 (19)	0.58914 (15)	0.0619 (6)	
C26	0.4247 (2)	0.26458 (17)	0.54571 (14)	0.0540 (5)	
H26A	0.5201	0.2753	0.5468	0.065*	
C27	0.4069 (2)	0.17962 (16)	0.49972 (12)	0.0457 (4)	
H27A	0.4918	0.1332	0.4694	0.055*	
C28	0.1336 (2)	−0.18155 (14)	0.07132 (12)	0.0394 (4)	
C29	0.1885 (2)	−0.22550 (17)	−0.01543 (13)	0.0551 (5)	
H29A	0.2832	−0.2131	−0.0543	0.066*	
C30	0.1038 (3)	−0.28786 (19)	−0.04489 (18)	0.0736 (7)	
H30A	0.1420	−0.3172	−0.1031	0.088*	
C31	−0.0349 (3)	−0.30622 (19)	0.0110 (2)	0.0766 (7)	
H31A	−0.0904	−0.3495	−0.0084	0.092*	
C32	−0.0932 (3)	−0.2612 (2)	0.09581 (17)	0.0706 (6)	
H32A	−0.1889	−0.2725	0.1333	0.085*	
C33	−0.0093 (2)	−0.19859 (18)	0.12561 (14)	0.0551 (5)	
H33A	−0.0497	−0.1676	0.1829	0.066*	
C34	0.34205 (19)	−0.29458 (13)	0.23043 (11)	0.0361 (4)	
C35	0.4672 (2)	−0.33131 (15)	0.16058 (13)	0.0463 (4)	
H35A	0.5004	−0.2780	0.1029	0.056*	
C36	0.5438 (2)	−0.44564 (16)	0.17465 (16)	0.0561 (5)	
H36A	0.6284	−0.4692	0.1277	0.067*	
C37	0.4923 (2)	−0.52266 (15)	0.25895 (17)	0.0574 (5)	
C38	0.3723 (3)	−0.49040 (17)	0.33078 (16)	0.0604 (5)	
H38A	0.3414	−0.5444	0.3885	0.073*	
C39	0.2972 (2)	−0.37606 (15)	0.31647 (13)	0.0485 (4)	
H39A	0.2150	−0.3531	0.3653	0.058*	
H1O2	0.334 (2)	0.0994 (18)	0.0207 (15)	0.068 (7)*	
H1N2	0.078 (2)	0.0265 (17)	0.1045 (15)	0.063 (6)*	

### Atomic displacement parameters (Å^2^)

**Table:**

	*U*^11^	*U*^22^	*U*^33^	*U*^12^	*U*^13^	*U*^23^
F1	0.1084 (12)	0.1056 (11)	0.1283 (12)	−0.0232 (9)	−0.0316 (10)	−0.0792 (10)
F2	0.0824 (10)	0.0379 (6)	0.1443 (12)	0.0081 (6)	−0.0506 (9)	−0.0034 (7)
O1	0.0685 (9)	0.0515 (7)	0.0319 (6)	−0.0280 (6)	−0.0041 (6)	−0.0042 (5)
O2	0.0559 (8)	0.0511 (7)	0.0276 (6)	−0.0205 (6)	0.0018 (5)	−0.0083 (5)
N1	0.0367 (8)	0.0365 (7)	0.0340 (7)	−0.0090 (6)	−0.0025 (6)	−0.0097 (5)
N2	0.0495 (9)	0.0322 (7)	0.0346 (7)	−0.0078 (7)	−0.0143 (6)	−0.0067 (5)
C1	0.0363 (9)	0.0351 (8)	0.0293 (7)	−0.0041 (7)	−0.0074 (7)	−0.0062 (6)
C2	0.0418 (10)	0.0445 (10)	0.0446 (9)	−0.0072 (8)	−0.0053 (8)	−0.0054 (8)
C3	0.0398 (11)	0.0667 (13)	0.0510 (11)	−0.0010 (9)	0.0002 (9)	−0.0138 (9)
C4	0.0485 (12)	0.0602 (13)	0.0569 (11)	0.0106 (10)	−0.0112 (9)	−0.0270 (9)
C5	0.0515 (11)	0.0416 (10)	0.0458 (9)	0.0031 (8)	−0.0189 (8)	−0.0194 (8)
C6	0.0693 (14)	0.0393 (10)	0.0703 (13)	0.0041 (10)	−0.0281 (11)	−0.0247 (9)
C7	0.0788 (16)	0.0332 (10)	0.0742 (13)	−0.0109 (10)	−0.0338 (12)	−0.0117 (9)
C8	0.0576 (12)	0.0374 (10)	0.0532 (10)	−0.0153 (9)	−0.0162 (9)	−0.0075 (8)
C9	0.0470 (10)	0.0334 (8)	0.0338 (8)	−0.0086 (7)	−0.0122 (7)	−0.0073 (6)
C10	0.0439 (10)	0.0344 (8)	0.0322 (8)	−0.0044 (7)	−0.0127 (7)	−0.0087 (6)
C11	0.0393 (9)	0.0351 (8)	0.0281 (7)	−0.0104 (7)	−0.0012 (7)	−0.0076 (6)
C13	0.0413 (9)	0.0325 (8)	0.0317 (8)	−0.0078 (7)	−0.0060 (7)	−0.0080 (6)
C14	0.0366 (9)	0.0332 (8)	0.0307 (7)	−0.0083 (7)	−0.0056 (6)	−0.0074 (6)
C15	0.0354 (9)	0.0300 (8)	0.0291 (7)	−0.0066 (7)	−0.0052 (6)	−0.0063 (6)
C16	0.0390 (9)	0.0296 (8)	0.0290 (7)	−0.0085 (7)	−0.0059 (6)	−0.0056 (6)
C17	0.0358 (9)	0.0348 (8)	0.0311 (8)	−0.0061 (7)	−0.0070 (7)	−0.0047 (6)
C18	0.0372 (9)	0.0362 (8)	0.0307 (8)	−0.0071 (7)	−0.0087 (7)	−0.0056 (6)
C19	0.0381 (9)	0.0423 (9)	0.0369 (8)	−0.0115 (7)	−0.0075 (7)	−0.0102 (7)
C20	0.0374 (9)	0.0366 (9)	0.0364 (8)	−0.0048 (7)	−0.0037 (7)	−0.0101 (7)
C21	0.0435 (10)	0.0435 (9)	0.0339 (8)	−0.0133 (8)	−0.0071 (7)	−0.0069 (7)
C22	0.0447 (10)	0.0454 (10)	0.0301 (8)	−0.0108 (8)	−0.0043 (7)	−0.0107 (7)
C23	0.0445 (11)	0.0833 (15)	0.0718 (13)	−0.0175 (11)	0.0027 (10)	−0.0456 (12)
C24	0.0604 (14)	0.0917 (17)	0.0944 (17)	−0.0149 (13)	0.0037 (12)	−0.0661 (14)
C25	0.0756 (15)	0.0627 (13)	0.0626 (12)	−0.0189 (11)	−0.0156 (11)	−0.0335 (10)
C26	0.0565 (12)	0.0600 (12)	0.0558 (11)	−0.0145 (10)	−0.0243 (10)	−0.0136 (9)
C27	0.0448 (10)	0.0504 (11)	0.0451 (9)	−0.0045 (8)	−0.0149 (8)	−0.0130 (8)
C28	0.0511 (11)	0.0321 (8)	0.0381 (8)	−0.0064 (7)	−0.0169 (8)	−0.0055 (7)
C29	0.0700 (14)	0.0553 (12)	0.0475 (10)	−0.0040 (10)	−0.0224 (10)	−0.0192 (9)
C30	0.109 (2)	0.0606 (14)	0.0721 (14)	−0.0011 (14)	−0.0513 (15)	−0.0295 (11)
C31	0.113 (2)	0.0535 (13)	0.0912 (17)	−0.0260 (14)	−0.0699 (17)	−0.0037 (12)
C32	0.0741 (15)	0.0735 (15)	0.0764 (15)	−0.0347 (12)	−0.0385 (13)	0.0060 (12)
C33	0.0587 (13)	0.0617 (12)	0.0526 (11)	−0.0207 (10)	−0.0174 (10)	−0.0107 (9)
C34	0.0414 (9)	0.0313 (8)	0.0395 (8)	−0.0102 (7)	−0.0120 (7)	−0.0074 (7)
C35	0.0485 (11)	0.0342 (9)	0.0546 (10)	−0.0083 (8)	−0.0073 (9)	−0.0087 (8)
C36	0.0456 (11)	0.0436 (11)	0.0806 (14)	−0.0014 (9)	−0.0146 (10)	−0.0195 (10)
C37	0.0573 (13)	0.0317 (10)	0.0896 (15)	−0.0016 (9)	−0.0389 (12)	−0.0045 (10)
C38	0.0750 (15)	0.0423 (11)	0.0630 (12)	−0.0126 (10)	−0.0296 (11)	0.0096 (9)
C39	0.0589 (12)	0.0414 (10)	0.0442 (9)	−0.0119 (9)	−0.0133 (9)	−0.0013 (8)

### Geometric parameters (Å, °)

**Table:**

F1—C25	1.353 (2)	C17—C18	1.495 (2)
F2—C37	1.362 (2)	C18—C21	1.336 (2)
O1—C17	1.2181 (18)	C18—C19	1.530 (2)
O2—C11	1.4110 (17)	C19—H19A	0.9700
O2—H1O2	0.90 (2)	C19—H19B	0.9700
N1—C20	1.466 (2)	C20—H20A	0.9700
N1—C11	1.475 (2)	C20—H20B	0.9700
N1—C19	1.4781 (19)	C21—C22	1.467 (2)
N2—C16	1.4675 (19)	C21—H21A	0.9300
N2—C13	1.484 (2)	C22—C27	1.386 (2)
N2—H1N2	0.91 (2)	C22—C23	1.387 (2)
C1—C2	1.366 (2)	C23—C24	1.380 (3)
C1—C10	1.408 (2)	C23—H23A	0.9300
C1—C16	1.516 (2)	C24—C25	1.363 (3)
C2—C3	1.416 (2)	C24—H24A	0.9300
C2—H2A	0.9300	C25—C26	1.357 (3)
C3—C4	1.359 (3)	C26—C27	1.386 (2)
C3—H3A	0.9300	C26—H26A	0.9300
C4—C5	1.418 (3)	C27—H27A	0.9300
C4—H4A	0.9300	C28—C33	1.382 (3)
C5—C10	1.408 (2)	C28—C29	1.386 (2)
C5—C6	1.410 (3)	C29—C30	1.387 (3)
C6—C7	1.370 (3)	C29—H29A	0.9300
C6—H6A	0.9300	C30—C31	1.359 (3)
C7—C8	1.412 (3)	C30—H30A	0.9300
C7—H7A	0.9300	C31—C32	1.371 (3)
C8—C9	1.371 (2)	C31—H31A	0.9300
C8—H8A	0.9300	C32—C33	1.386 (3)
C9—C10	1.398 (2)	C32—H32A	0.9300
C9—C11	1.508 (2)	C33—H33A	0.9300
C11—C16	1.601 (2)	C34—C35	1.385 (2)
C13—C28	1.506 (2)	C34—C39	1.390 (2)
C13—C14	1.547 (2)	C35—C36	1.385 (2)
C13—H13A	0.9800	C35—H35A	0.9300
C14—C34	1.517 (2)	C36—C37	1.361 (3)
C14—C15	1.526 (2)	C36—H36A	0.9300
C14—H14A	0.9800	C37—C38	1.359 (3)
C15—C17	1.509 (2)	C38—C39	1.381 (3)
C15—C20	1.552 (2)	C38—H38A	0.9300
C15—C16	1.570 (2)	C39—H39A	0.9300
			
C11—O2—H1O2	104.9 (13)	C21—C18—C19	125.49 (15)
C20—N1—C11	102.48 (12)	C17—C18—C19	118.04 (12)
C20—N1—C19	108.12 (12)	N1—C19—C18	115.25 (13)
C11—N1—C19	115.60 (12)	N1—C19—H19A	108.5
C16—N2—C13	109.96 (12)	C18—C19—H19A	108.5
C16—N2—H1N2	111.2 (13)	N1—C19—H19B	108.5
C13—N2—H1N2	113.2 (13)	C18—C19—H19B	108.5
C2—C1—C10	119.20 (15)	H19A—C19—H19B	107.5
C2—C1—C16	131.77 (15)	N1—C20—C15	104.08 (12)
C10—C1—C16	108.91 (13)	N1—C20—H20A	110.9
C1—C2—C3	118.44 (17)	C15—C20—H20A	110.9
C1—C2—H2A	120.8	N1—C20—H20B	110.9
C3—C2—H2A	120.8	C15—C20—H20B	110.9
C4—C3—C2	122.67 (18)	H20A—C20—H20B	109.0
C4—C3—H3A	118.7	C18—C21—C22	129.62 (16)
C2—C3—H3A	118.7	C18—C21—H21A	115.2
C3—C4—C5	120.48 (16)	C22—C21—H21A	115.2
C3—C4—H4A	119.8	C27—C22—C23	117.87 (16)
C5—C4—H4A	119.8	C27—C22—C21	123.22 (15)
C10—C5—C6	115.95 (17)	C23—C22—C21	118.81 (16)
C10—C5—C4	116.03 (16)	C24—C23—C22	120.89 (19)
C6—C5—C4	127.95 (17)	C24—C23—H23A	119.6
C7—C6—C5	120.74 (17)	C22—C23—H23A	119.6
C7—C6—H6A	119.6	C25—C24—C23	119.03 (19)
C5—C6—H6A	119.6	C25—C24—H24A	120.5
C6—C7—C8	122.24 (18)	C23—C24—H24A	120.5
C6—C7—H7A	118.9	F1—C25—C26	119.1 (2)
C8—C7—H7A	118.9	F1—C25—C24	118.54 (19)
C9—C8—C7	118.34 (18)	C26—C25—C24	122.37 (18)
C9—C8—H8A	120.8	C25—C26—C27	118.26 (18)
C7—C8—H8A	120.8	C25—C26—H26A	120.9
C8—C9—C10	119.39 (16)	C27—C26—H26A	120.9
C8—C9—C11	132.10 (16)	C26—C27—C22	121.57 (17)
C10—C9—C11	108.41 (13)	C26—C27—H27A	119.2
C9—C10—C5	123.22 (16)	C22—C27—H27A	119.2
C9—C10—C1	113.65 (14)	C33—C28—C29	118.09 (17)
C5—C10—C1	123.03 (16)	C33—C28—C13	122.14 (15)
O2—C11—N1	107.74 (12)	C29—C28—C13	119.71 (17)
O2—C11—C9	112.93 (12)	C28—C29—C30	120.6 (2)
N1—C11—C9	115.59 (12)	C28—C29—H29A	119.7
O2—C11—C16	109.11 (12)	C30—C29—H29A	119.7
N1—C11—C16	106.18 (11)	C31—C30—C29	120.3 (2)
C9—C11—C16	104.93 (12)	C31—C30—H30A	119.9
N2—C13—C28	113.74 (14)	C29—C30—H30A	119.9
N2—C13—C14	104.58 (11)	C30—C31—C32	120.2 (2)
C28—C13—C14	114.42 (13)	C30—C31—H31A	119.9
N2—C13—H13A	107.9	C32—C31—H31A	119.9
C28—C13—H13A	107.9	C31—C32—C33	119.9 (2)
C14—C13—H13A	107.9	C31—C32—H32A	120.1
C34—C14—C15	117.75 (13)	C33—C32—H32A	120.1
C34—C14—C13	114.81 (12)	C28—C33—C32	120.91 (19)
C15—C14—C13	102.44 (11)	C28—C33—H33A	119.5
C34—C14—H14A	107.1	C32—C33—H33A	119.5
C15—C14—H14A	107.1	C35—C34—C39	117.66 (16)
C13—C14—H14A	107.1	C35—C34—C14	122.94 (14)
C17—C15—C14	116.38 (12)	C39—C34—C14	119.31 (15)
C17—C15—C20	106.73 (12)	C36—C35—C34	121.48 (17)
C14—C15—C20	117.62 (12)	C36—C35—H35A	119.3
C17—C15—C16	108.69 (12)	C34—C35—H35A	119.3
C14—C15—C16	104.15 (12)	C37—C36—C35	118.36 (19)
C20—C15—C16	101.98 (12)	C37—C36—H36A	120.8
N2—C16—C1	112.71 (13)	C35—C36—H36A	120.8
N2—C16—C15	105.47 (12)	C38—C37—C36	122.46 (17)
C1—C16—C15	119.60 (12)	C38—C37—F2	118.92 (19)
N2—C16—C11	112.65 (12)	C36—C37—F2	118.6 (2)
C1—C16—C11	103.30 (12)	C37—C38—C39	118.78 (18)
C15—C16—C11	102.82 (12)	C37—C38—H38A	120.6
O1—C17—C18	122.52 (13)	C39—C38—H38A	120.6
O1—C17—C15	122.42 (14)	C38—C39—C34	121.21 (18)
C18—C17—C15	115.02 (13)	C38—C39—H39A	119.4
C21—C18—C17	116.22 (14)	C34—C39—H39A	119.4
			
C10—C1—C2—C3	−1.3 (2)	N1—C11—C16—N2	−124.04 (13)
C16—C1—C2—C3	−176.77 (16)	C9—C11—C16—N2	113.09 (14)
C1—C2—C3—C4	2.8 (3)	O2—C11—C16—C1	−130.08 (13)
C2—C3—C4—C5	−0.7 (3)	N1—C11—C16—C1	114.06 (12)
C3—C4—C5—C10	−2.7 (3)	C9—C11—C16—C1	−8.81 (14)
C3—C4—C5—C6	174.06 (18)	O2—C11—C16—C15	104.87 (13)
C10—C5—C6—C7	−0.7 (3)	N1—C11—C16—C15	−11.00 (14)
C4—C5—C6—C7	−177.53 (18)	C9—C11—C16—C15	−133.86 (12)
C5—C6—C7—C8	−1.7 (3)	C14—C15—C17—O1	−2.3 (2)
C6—C7—C8—C9	1.6 (3)	C20—C15—C17—O1	−135.86 (16)
C7—C8—C9—C10	0.9 (2)	C16—C15—C17—O1	114.85 (17)
C7—C8—C9—C11	176.86 (17)	C14—C15—C17—C18	179.67 (13)
C8—C9—C10—C5	−3.5 (2)	C20—C15—C17—C18	46.08 (17)
C11—C9—C10—C5	179.68 (14)	C16—C15—C17—C18	−63.21 (17)
C8—C9—C10—C1	173.13 (14)	O1—C17—C18—C21	−24.6 (2)
C11—C9—C10—C1	−3.69 (18)	C15—C17—C18—C21	153.42 (15)
C6—C5—C10—C9	3.3 (2)	O1—C17—C18—C19	160.88 (15)
C4—C5—C10—C9	−179.46 (15)	C15—C17—C18—C19	−21.1 (2)
C6—C5—C10—C1	−172.97 (15)	C20—N1—C19—C18	−47.68 (17)
C4—C5—C10—C1	4.2 (2)	C11—N1—C19—C18	66.45 (17)
C2—C1—C10—C9	−178.85 (14)	C21—C18—C19—N1	−153.25 (16)
C16—C1—C10—C9	−2.45 (18)	C17—C18—C19—N1	20.7 (2)
C2—C1—C10—C5	−2.2 (2)	C11—N1—C20—C15	−48.50 (13)
C16—C1—C10—C5	174.19 (14)	C19—N1—C20—C15	74.05 (15)
C20—N1—C11—O2	−80.31 (13)	C17—C15—C20—N1	−73.35 (14)
C19—N1—C11—O2	162.36 (12)	C14—C15—C20—N1	153.74 (12)
C20—N1—C11—C9	152.34 (13)	C16—C15—C20—N1	40.60 (14)
C19—N1—C11—C9	35.01 (18)	C17—C18—C21—C22	179.54 (16)
C20—N1—C11—C16	36.48 (13)	C19—C18—C21—C22	−6.4 (3)
C19—N1—C11—C16	−80.86 (14)	C18—C21—C22—C27	−40.3 (3)
C8—C9—C11—O2	−49.7 (2)	C18—C21—C22—C23	143.4 (2)
C10—C9—C11—O2	126.53 (14)	C27—C22—C23—C24	0.8 (3)
C8—C9—C11—N1	75.0 (2)	C21—C22—C23—C24	177.3 (2)
C10—C9—C11—N1	−108.77 (15)	C22—C23—C24—C25	−0.9 (4)
C8—C9—C11—C16	−168.46 (17)	C23—C24—C25—F1	−179.9 (2)
C10—C9—C11—C16	7.80 (16)	C23—C24—C25—C26	0.4 (4)
C16—N2—C13—C28	148.04 (13)	F1—C25—C26—C27	−179.51 (18)
C16—N2—C13—C14	22.51 (17)	C24—C25—C26—C27	0.2 (3)
N2—C13—C14—C34	−164.10 (13)	C25—C26—C27—C22	−0.2 (3)
C28—C13—C14—C34	70.80 (18)	C23—C22—C27—C26	−0.3 (3)
N2—C13—C14—C15	−35.25 (16)	C21—C22—C27—C26	−176.57 (16)
C28—C13—C14—C15	−160.35 (13)	N2—C13—C28—C33	−69.3 (2)
C34—C14—C15—C17	−78.91 (17)	C14—C13—C28—C33	50.8 (2)
C13—C14—C15—C17	154.11 (13)	N2—C13—C28—C29	113.44 (17)
C34—C14—C15—C20	49.57 (18)	C14—C13—C28—C29	−126.44 (16)
C13—C14—C15—C20	−77.41 (16)	C33—C28—C29—C30	−1.9 (3)
C34—C14—C15—C16	161.50 (12)	C13—C28—C29—C30	175.42 (17)
C13—C14—C15—C16	34.52 (14)	C28—C29—C30—C31	0.3 (3)
C13—N2—C16—C1	−132.84 (14)	C29—C30—C31—C32	1.3 (3)
C13—N2—C16—C15	−0.66 (16)	C30—C31—C32—C33	−1.2 (3)
C13—N2—C16—C11	110.74 (14)	C29—C28—C33—C32	2.1 (3)
C2—C1—C16—N2	60.9 (2)	C13—C28—C33—C32	−175.23 (17)
C10—C1—C16—N2	−114.87 (14)	C31—C32—C33—C28	−0.5 (3)
C2—C1—C16—C15	−63.9 (2)	C15—C14—C34—C35	−85.00 (19)
C10—C1—C16—C15	120.36 (14)	C13—C14—C34—C35	35.7 (2)
C2—C1—C16—C11	−177.22 (16)	C15—C14—C34—C39	98.43 (18)
C10—C1—C16—C11	7.00 (15)	C13—C14—C34—C39	−140.82 (16)
C17—C15—C16—N2	−146.38 (13)	C39—C34—C35—C36	1.1 (3)
C14—C15—C16—N2	−21.70 (15)	C14—C34—C35—C36	−175.49 (16)
C20—C15—C16—N2	101.13 (13)	C34—C35—C36—C37	0.7 (3)
C17—C15—C16—C1	−18.22 (19)	C35—C36—C37—C38	−2.3 (3)
C14—C15—C16—C1	106.46 (15)	C35—C36—C37—F2	176.64 (16)
C20—C15—C16—C1	−130.71 (14)	C36—C37—C38—C39	1.9 (3)
C17—C15—C16—C11	95.40 (13)	F2—C37—C38—C39	−177.05 (17)
C14—C15—C16—C11	−139.92 (11)	C37—C38—C39—C34	0.1 (3)
C20—C15—C16—C11	−17.09 (13)	C35—C34—C39—C38	−1.6 (3)
O2—C11—C16—N2	−8.18 (18)	C14—C34—C39—C38	175.19 (16)

### Hydrogen-bond geometry (Å, °)

**Table:**

Cg5, Cg6, Cg7 and Cg9 are the centroids of the C1–C5/C10, C5–C10, C22–C27 and C34–C39 rings, respectively.

**Table:**

*D*—H···*A*	*D*—H	H···*A*	*D*···*A*	*D*—H···*A*
O2—H1O2···N2	0.90 (2)	1.97 (2)	2.636 (2)	129.6 (17)
C13—H13A···O2^i^	0.98	2.53	3.499 (2)	171
C20—H20B···O2^i^	0.97	2.55	3.522 (2)	179
C23—H23A···O1^ii^	0.93	2.52	3.410 (2)	162
C35—H35A···O2^i^	0.93	2.46	3.383 (2)	174
C30—H30A···Cg5^iii^	0.93	2.94	3.836 (2)	168
C31—H31A···Cg6^iii^	0.93	2.91	3.609 (3)	133
C38—H38A···Cg7^iv^	0.93	2.92	3.836 (2)	168
C7—H7A···Cg9^v^	0.93	2.85	3.400 (3)	119

Symmetry codes: (i) −*x*+1, −*y*, −*z*; (ii) −*x*, −*y*, −*z*+1; (iii) −*x*, −*y*, −*z*; (iv) *x*, *y*−1, *z*; (v) *x*, *y*+1, *z*.
